# Comparison of suprapatellar versus infrapatellar approaches of intramedullary nailing for distal tibia fractures

**DOI:** 10.1186/s13018-020-01960-8

**Published:** 2020-09-17

**Authors:** Yao Lu, Gen Wang, Bin Hu, Cheng Ren, Liang Sun, Zhimeng Wang, Changjun He, Hanzhong Xue, Zhong Li, Kun Zhang, Teng Ma, Qian Wang

**Affiliations:** 1 Department of Orthopaedic Surgery, HongHui Hospital, Xi’an Jiaotong University, 555 Youyi East Road, Xi’an, 710054 Shaan’xi Province China; 2 Bioinspired Engineering and Biomechanics Center (BEBC), School of Life Science and Technology, Xi’an Jiaotong University, Xi’an, 710049 China; 3 Orthopaedics Institute of Chinese PLA, 80th Hospital, 3770 Beigongxijie, Weifang, Shandong Province China; 4 Department of Hematology, Xi’an Gao Xin Hospital, Xi’an, 710054 Shaan’xi Province China; 5 Yan’an University, Yan’an, 710000 Shaanxi China

**Keywords:** Distal tibia fracture, Intramedullary nail, Internal fixation

## Abstract

**Background:**

This study aimed to analyze and compare the clinical and functional outcomes of distal tibia fractures treated with intramedullary nailing (IMN) using the suprapatellar (SP) and infrapatellar (IP) surgical approaches.

**Methods:**

A retrospective analysis was performed in 63 patients with distal fractures that were treated with IMN between August 2014 and August 2018. A total of 27 and 36 patients underwent IMN using the SP and IP techniques, respectively. The surgical time, blood loss, closed reduction rate, rate of adjuvant reduction technique, fracture healing time, and complications were reviewed in this study. Anterior knee pain was assessed using the visual analog scale. The Lysholm Knee Scoring Scale and American Orthopaedic Foot and Ankle Society (AOFAS) scale were used as clinical measurements.

**Results:**

A total of 63 patients, with a minimum follow-up of 12 months, were evaluated. The average surgical time, blood loss, rate of adjuvant reduction technique, closed reduction rate, fracture healing time, and Lysholm Knee Scoring Scale score were insignificantly different (*P* > 0.05) between the two groups. However, the SP approach was superior to the IP approach in terms of pain score, AOFAS score, and fracture deformity rate (*P* < 0.05).

**Conclusions:**

In the treatment of distal tibia fractures, the SP IMN technique is associated with a significantly higher functional outcome, lower knee pain, and lower rate of fracture deformity than the IP IMN technique.

## Background

Distal tibia fracture is a common clinical wound that usually results from high-energy injuries [1, 2]. Open reduction and internal fixation with plates and screws is the common method to treat distal tibia fractures [3–5]. However, plate fixation management of these fractures has often resulted in complications such as infections, delayed unions or nonunions, and implant failures [6–8]. In recent years, intramedullary nailing (IMN) and minimally invasive plate osteosynthesis (MIPO) have become common fixation methods for distal tibia fractures [9, 10]. Our previous study of a meta-analysis based on 13 randomized controlled trials (RCTs) with 924 patients revealed that IMN for distal tibia fractures is associated with a lower risk of wound complications and a shorter time to union than those for MIPO [11]. IMN insertion comprises the traditional infrapatellar (IP) approach and suprapatellar (SP) approach in the semiextended position. On the basis of the clinical outcomes, several studies showed that the SP and IP approaches have similar functional outcomes for tibial shaft fractures. However, valid evidence confirming the effectiveness of both approaches in treating distal tibial fractures is insufficient.

This study aimed to compare the clinical and functional outcomes of distal tibia fractures treated with tibial nailing using the SP and IP surgical approaches.

## Methods

This retrospective review was conducted at a level-one trauma center of Honghui Hospital, Xi’an Jiaotong University College of Medicine. Skeletally mature patients with distal tibial metadiaphyseal fractures who underwent treatment with tibial intramedullary nails between August 2014 and August 2018 were identified. The distal tibia fracture was defined as a fracture with its major fracture line located 12 cm above the medial to lateral width of the articular surface of the ankle. The distal tibia fracture was graded according to the AO Foundation/Orthopaedic Trauma Association (OTA/AO) classification scheme based on the initial injury films and computed tomography scans. The inclusion criteria were as follows: extraarticular tibia fractures (OTA 43-A), nondisplaced intraarticular fractures (OTA 43-C1 and OTA 43-C2), and fractures with major fracture lines located within 12 cm the distal tibial plafond. The exclusion criteria were as follows: old distal tibia fracture, an ipsilateral knee injury, severe ankle diseases such as preoperative rheumatoid arthritis and gouty arthritis, and insufficient chart or radiographic data.

Patients were divided into the following two groups: patients treated using an IP IMN insertion technique and patients treating using an SP IMN technique. All surgeries were performed by the senior orthopedic surgeons who were well trained in both techniques. General anesthesia or spinal-epidural anesthesia was induced, and patients were placed in the supine position with their lesioned-side hip elevated. A pneumatic tourniquet was used routinely at the thigh region, adapting a pressure of 60 kPa. For patients complicated with fibula fractures where the fracture lines were within 8 cm above the malleolar fossa, the locking plate or 1/3 tube plate was adapted first via the lateral approach to fix the distal fibula, and the temporary full-thickness suture was used to maintain skin tension.

Regarding the IP approach group, the patellar ligament was split in the middle from the prepatellar midline approach with the knee flexed to approximately 90°. A hole was opened at the slope along the intramedullary cavity using a device, while traction and reduction were performed by the surgeon’s assistant. Moreover, the C-arm X-ray imaging system after the guide wire insertion was used to assess the fracture position and alignment. Upon successful completion of the fracture reduction, suitable intramedullary nails were inserted along the guide wire; hence, the nail tip was maximally close to the articular surface of the distal tibia. The fracture position and alignment were evaluated using the C-arm X-ray imaging system. When a fracture is difficult to reset, a blocking nail technique and a reduction clamp can be used to assist in the reduction. After achieving the satisfactory reduction, the fracture was fixed using proximal and distal locking screws.

**Fig. 1:**
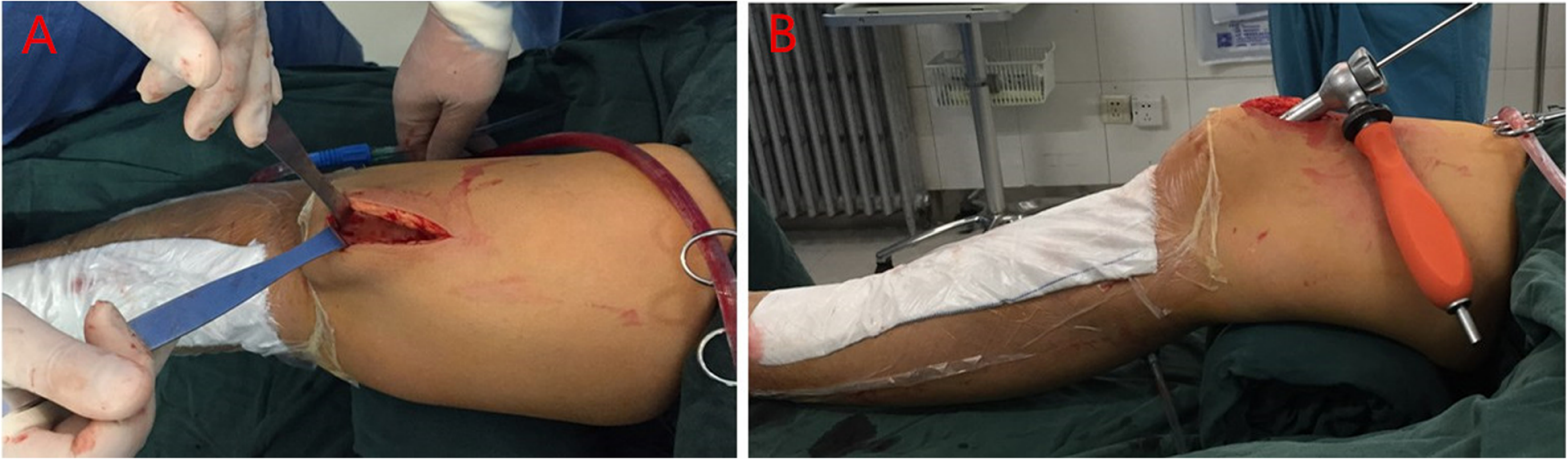
**a** Suprapatellar skin incision approximately 3 cm proximal to the superior pole of the patella. **b** Protective trocar placement with the knee in the semiextended position

**Fig. 2:**
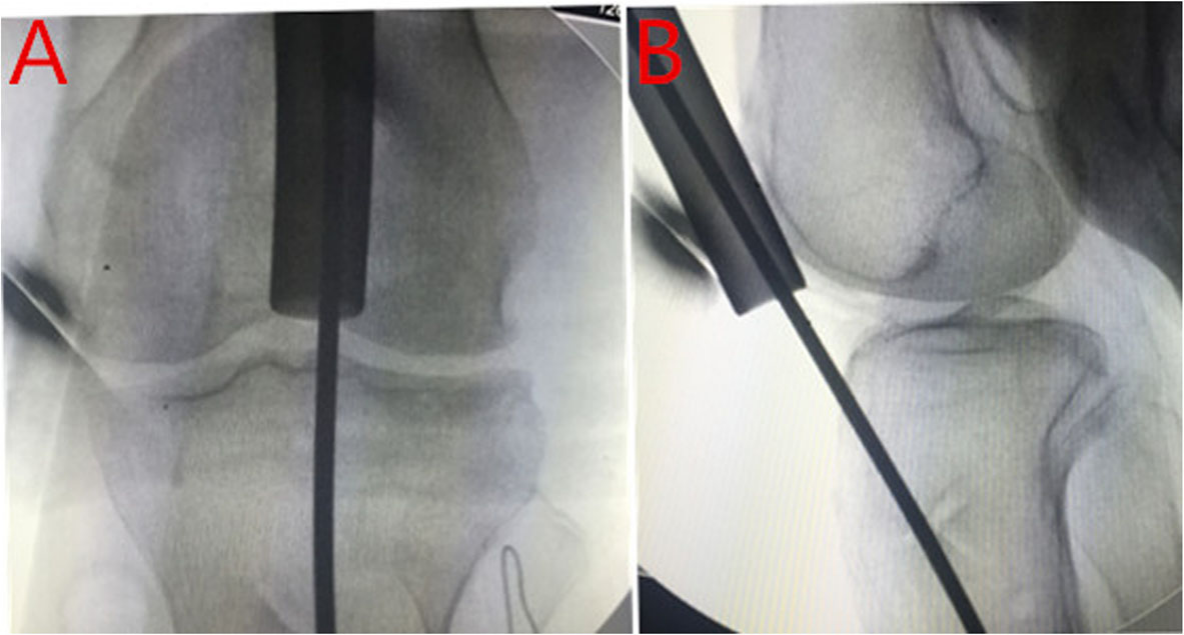
Starting point under fluoroscopic guidance

**Fig. 3:**
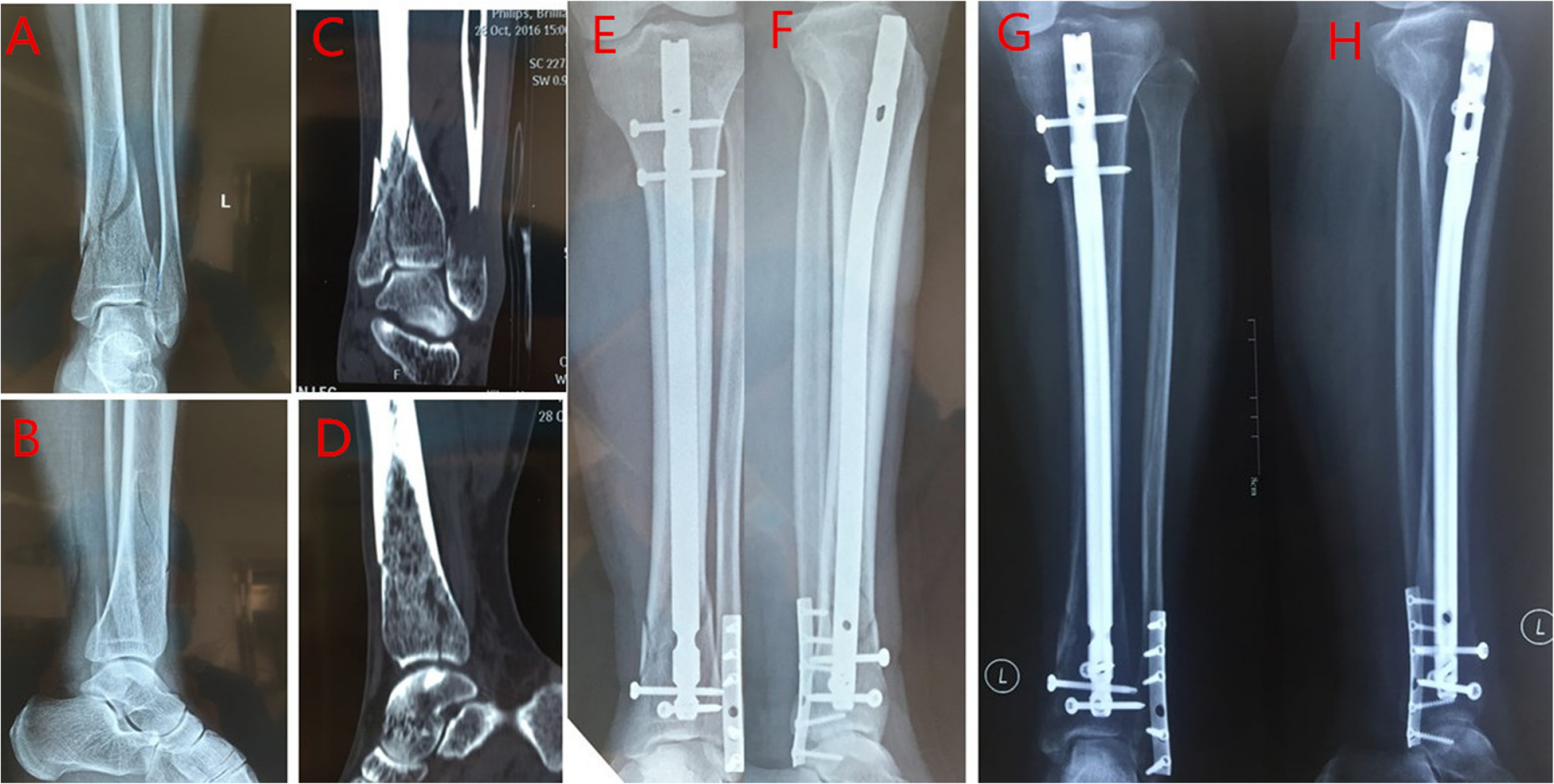
Radiographs of a case of union after closed reduction using the suprapatellar approach were presented. **a**, **b** Preoperative anteroposterior (AP) and lateral views. **c**, **d** Computed tomography views, fracture involving the ankle joint. **e**, **f** AP and lateral views postoperatively. **g**, **h** AP and lateral views 6 months postoperatively

**Fig. 4:**
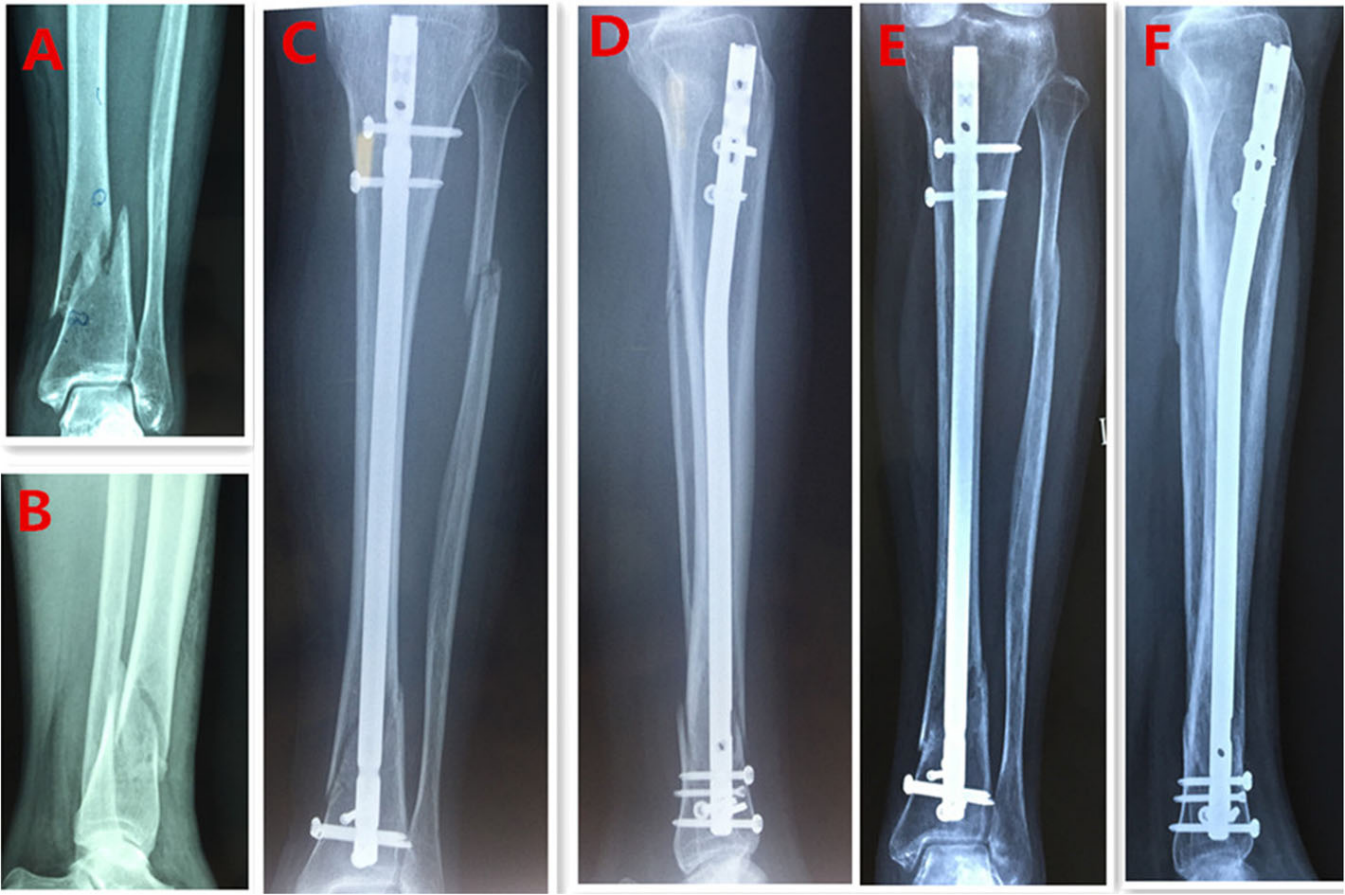
Radiographs of a case of union after closed reduction using the infrapatellar approach were presented. **a**, **b** Preoperative anteroposterior (AP) and lateral views. **c**, **d** AP and lateral views postoperatively. **e**, **f** AP and lateral views 5 months postoperatively

Regarding the SP approach group (Fig. 1), a 3-cm incision was made proximal to the superior pole of the patella. The knee was positioned in 20–30° flexion. The quadriceps tendon and articular capsule were dissected lengthwise. A specialized SP insertion cannula within a protective sleeve was placed through the skin incision, through the trochlear groove under the surface of the patella, and at the desired start point for tibial nailing, which is in the intersection of tibial midline and tibial plateau articular surface (Fig. 2). The position of entry point was determined with the guidance of C-arm. Subsequently, IMN was performed using a cannula-sleeve device as per convention. Radiographs of a case of union after closed reduction using the suprapatellar approach were presented (Fig. 3). Radiographs of a case of union after closed reduction using the infrapatellar approach were presented (Fig. 4). Postoperative radiography was routinely performed (Figs. 1, 2, 3, and 4).

All patients were contacted at a minimum of 12 months following surgery for a clinical and radiological follow-up. The ankle outcomes of all patients were evaluated by a trained and experienced orthopedic surgeon using the guidelines of the American Orthopaedic Foot and Ankle Society (AOFAS) scale [12], and the knee outcomes of all patients were evaluated using the Lysholm Knee Scoring Scale [13]. Patients’ pain scores using the visual analog scale (VAS) were recorded. Coronal and sagittal alignments were evaluated by measuring the anatomical axis of the tibia on standard views. Fracture deformity was defined as greater than 5° in either the coronal or sagittal plane [14].

Statistical analyses were performed using GraphPad Prism version 8.0. The data were initially measured using the Shapiro-Wilk test to determine whether the data were normally distributed. The data of the Lysholm score, AOFAS ankle score, and angular deformity were normally distributed, and the variance was homogeneous. Data are presented as mean ± standard deviation. Subsequently, an unpaired Student’s *t* test was used to compare the two groups. Chi-squared test was applied to compare the differences in VAS between the two groups. A *P* value < 0.05 was considered significant.

## Results

### Comparison of sociodemographic data between the two groups

A total of 63 patients meeting the inclusion criteria were included in this study. Moreover, 27 (42.8%) and 36 (57.2%) patients were treated with SP IMN and IP IMN techniques, respectively. Patients’ sociodemographic data, including age, sex, fracture type, time to surgery, and follow-up time, were evenly matched in the two groups (Table 1).

**Table 1:** Comparison of sociodemographic data between the two groups

Characteristics	Suprapatellar	Infrapatellar	*P*
Age (years)	42.6 (10.2)	40.6 (11.3)	0.471
Sex (M/F)	11/16	20/16	0.311
AO classification (43 A/43C1/43 C2)	(15/8/4)	(20/9/7)	0.856
Time to surgery (days)	3.2 (1.1)	3.5 (1.2)	0.313
Follow-up (months)	23.2 (7.4)	24.3 (8.6)	0.596

### Surgical comparison between the two groups

The mean surgical times were 86.3 ± 14.6 min and 97.1 ± 16.9 min in the SP group and the IP group IP, respectively (*P* = 0.010). The average blood loss volumes during surgery were 56.3 ± 14.6 ml and 60.5 ± 9.3 ml in the SP group and the IP group IP, respectively (*P* = 0.099). The rates of adjuvant reduction technique were 33.3% (9/27) and 38.9% (14/36) in the SP group and the IP group, respectively (*P* = 0.819). The closed reduction rates were 92.6% (25/27) and 83.3% (30/36) in the SP group and the IP group, respectively (*P* = 0.448) (Table 2).

**Table 2:** Surgical and prognostic comparison between the two groups

Characteristics	Suprapatellar	Infrapatellar	*P*
Surgical time (min)	86.3 (14.6)	97.1 (16.9)	0.01
Blood loss (ml)	56.3 (10.6)	60.5 (9.3)	0.099
Adjuvant reduction technique (cases)	9 (27)	14 (36)	0.603
Closed reduction rate	25 (27)	30 (36)	0.448
Fracture healing (weeks)	12.2 (3.6)	12.8 (4.1)	0.549
Pain score	20.6 (3.7)	28.1 (3.4)	< 0.001
Lysholm score	88.6 (4.9)	85.7 (6.8)	0.061
Fracture deformity (cases)	1 (27)	9 (36)	0.034
AOFAS score	93.5 (4.2)	87.8 (4.9)	< 0.001

### Prognostic comparison

The mean fracture healing times were 12.2 ± 3.6 weeks and 12.8 ± 4.1 weeks in the SP group and the IP group, respectively (*P* = 0.549). The Lysholm Knee Scoring Scale scores were 88.6 ± 4.9 and 85.7 ± 6.8 in the SP group and the IP group, respectively (*P* = 0.061). The AOFAS scores were 93.5 ± 4.2 and 87.8 ± 4.9 in the SP group and the IP group, respectively (*P* < 0.001) (Table 2). The rate of deformity healing was lower in the SP group (3.7% [1/27]) than in the IP group (25% [9/36]) (*P* = 0.034) (Table 2).

## Discussion

Surgical treatment of distal tibia fractures can be performed with several techniques using external fixators, plates, and nails. Performing IMN as a treatment for distal tibia fractures has been an increasing trend considering that the intramedullary nails result in minimal injuries to the surrounding soft tissues with low risk of malunion and superior biomechanical strength [15]. IMN insertion comprises the traditional IP approach and SP approach in the semiextended position. Several studies showed that the SP and IP approaches have similar functional outcomes for tibial shaft fractures [16, 17]. However, valid evidence confirming the effectiveness of both approaches in treating distal tibial fractures is insufficient. This study aimed to compare the clinical and functional outcomes of distal tibia fractures treated with IMN using the SP and IP surgical approaches. Results showed that the surgical time, blood loss, and closed reduction rate were similar in both the SP IMN and IP IMN groups. The rate of adjuvant reduction technique in the SP group was significantly lower than that in the IP group (1.8 ± 0.4 vs. 2.7 ± 0.7, *P* < 0.05). Our results are consistent with the result of a previous study comparing the surgical outcomes between the SP and IP approaches. Yiliang Cui [16] investigated 24 and 26 patients who underwent SP IMN and IP IMN, respectively, with a minimum follow-up of 15 months and found no significant difference regarding the surgical time and blood loss between the two groups. Similarly, a meta-analysis of RCTs indicated that there were no significant differences in the blood loss and surgical time between the SP and IP groups [17]. However, another meta-analysis of RCTs indicated that SP IMN was superior to IP IMN in terms of total blood loss [18], which is possibly attributed to the different surgical techniques used when treating distal tibia fractures, specifically during fracture reduction or insertion of the nails and screws.

This study demonstrated that the VAS pain score was significantly lower in the SP group than in the IP group. This finding is consistent with the finding observed in a multicenter clinical trial conducted by MacDonald et al. who compared the VAS scores between the IP and SP approaches in 95 patients and demonstrated that the SP IMN surgical approach is associated with lower postoperative anterior knee pain than that associated with the IP IMN surgical approach [19]. A recent meta-analysis indicated that the SP approach was associated with a significant reduction in the VAS scores [17]. Postoperative knee pain is a relevant issue after IP IMN. Anterior knee pain has been reported in 50–70% of patients with tibial fractures treated with IP IMN [20]. After removing the hardware, only 30% of patients experience pain relief. Postoperative knee pain is associated with iatrogenic damage to the saphenous nerve and access-related scar formation of the Hoffa fat pad and the patellar tendon [21]. Furthermore, with the SP approach, the intramedullary nails can be inserted through the quadriceps tendon, thus keeping the patella tendon intact. Hence, the SP approach can significantly reduce the post-nailing knee pain rates [22, 23].

The rate of malalignment in the SP group (4.8%, 2/42) was significantly lower than that in the IP group (14.3%, 8/56). Our results are consistent with the result of a previous study reporting the radiographical outcomes following the treatment of distal tibia fractures using the SP and IP approaches [24]. Frank R compared the radiographical outcomes following the treatment with IMN using the SP and IP approaches and reported that there was a 26.1% incidence of angular deformity greater than 5° when IMN insertion was performed using the IP approach. In contrast, a 3.8% incidence of malalignment when IMN insertion was performed using the SP approach was observed. Marco Stella reported 2.9% malalignment in tibia fractures treated with IMN using the SP approach [25]. In the IP approach, the pull of the quadriceps and the backward deviation of the intramedullary nail caused the flexion of the proximal segment, resulting in the anterior flexion deformity [26]. In the SP approach, considering the ability to maintain the leg in a static position and the knee at approximately 15 to 20° of flexion, IMN can easily access the appropriate starting point while maintaining a relaxed extensor mechanism [27]. Thus, maintaining the leg in a static position throughout the operation led to the improvement of distal tibia fractures when performing IMN [28].

Evaluation on the basis of the Lysholm Knee Scoring Scale score of the IP and SP approaches for the treatment of distal tibial fractures in this study showed similar results. Our results are consistent with the results of the previous reports that compared the functional knee outcomes between the IP and SP surgical approaches [16, 29]. However, our study demonstrated that the AOFAS score was significantly higher in the SP approach than in the IP approach. Most studies reported similar AOFAS outcomes in distal tibia fractures treated with IMN and minimally invasive percutaneous plate osteosynthesis. Moreover, our previous study of a meta-analysis on the basis of 13 RCTs with 924 patients indicated that there were no significant differences in the AOFAS outcomes between the IMN and minimally invasive percutaneous plate osteosynthesis groups [11]. However, in the current study, we initially used the AOFAS scores to evaluate the functional outcomes of distal tibia fractures using the SP and IP approaches. We found that patients with distal tibia fractures might have better outcomes when undergoing IMN using the SP approach than when using the IP approach. This may be attributed to the lower rate of malunion of distal tibial fractures with the SP approach than with the IP approach.

This study has some limitations. First, this was a retrospective single-center study with a small sample size. Hence, a large-scale prospective, randomized case-control study is required to evaluate the effectiveness of the SP approach. Second, the patellofemoral joint was evaluated by radiography. Magnetic resonance imaging or arthroscopy examination should be conducted to evaluate the cartilage changes postoperatively and at final follow-up. Moreover, our study confirmed the feasibility and safety of distal tibia fractures treated with IMN using the SP approach. However, a study with a longer follow-up time for a comprehensive comparison between the SP and IP approaches is required.

## Conclusion

In conclusion, our study compared the SP approach and the IP approach for the treatment of distal tibia fractures with IMN. The results demonstrated higher functional outcomes, lower knee pain, and lower rate of malalignment with the SP approach than with the IP approach. Hence, the SP approach of IMN has been considered an effective therapeutic approach for distal tibia fractures.

## Data Availability

All data analyzed in this study has been provided in the manuscript.

## Abbreviations

IMN: Intramedullary nailing
SP: Suprapatellar
IP: Infrapatellar
AOFAS: American Orthopaedic Foot and Ankle Society
MIPO: Minimally invasive plate osteosynthesis
RCTs: Randomized controlled trials
OTA/AO: The AO Foundation/Orthopaedic Trauma Association
VAS: The visual analog scale
